# A Case in Which Focal Convulsion Was the Initial Sign of Fatal Aortic Dissection

**DOI:** 10.1055/s-0039-3401023

**Published:** 2019-12-31

**Authors:** Youichi Yanagawa, Kouhei Ishikawa, Hiroki Nagasawa, Ikuto Takeuchi, Kei Jitsuiki, Shunsuke Madokoro, Akihiko Kondo, Hiromichi Ohsaka, Kazuhiko Omori

**Affiliations:** 1 Department of Acute Critical Care Medicine, Juntendo University, Shizuoka Hospital, Izunokuni City, Shizuoka, Japan

**Keywords:** focal convulsion, aortic dissection, thromboembolism

## Abstract

Focal convulsion as an initial sign of aortic dissection is extremely rare. Type A aortic dissection involves the aortic arch, which may result in seizure either through the extension of the dissection into the common carotid arteries or through thromboembolism or cerebral hypoperfusion. Physicians should perform whole body computed tomography to determine whether or not dissection is present when treating patients with convulsion and a high level of fibrin degradation products.

## Introduction

Patients with aortic dissection (AD) present with various complaints and symptoms, with the major complaints being severe chest and back pain, which can shift with the progression of AD. However, AD can be painless and lead to various symptoms, such as hoarseness, heart failure, syncope, stroke, paraplegia, anuria, or sudden death. 1 2 We, herein, present the case of a patient with acute aortic dissection Type A whose initial complaint was focal convulsion.

## Case Presentation

**Fig. 1:**
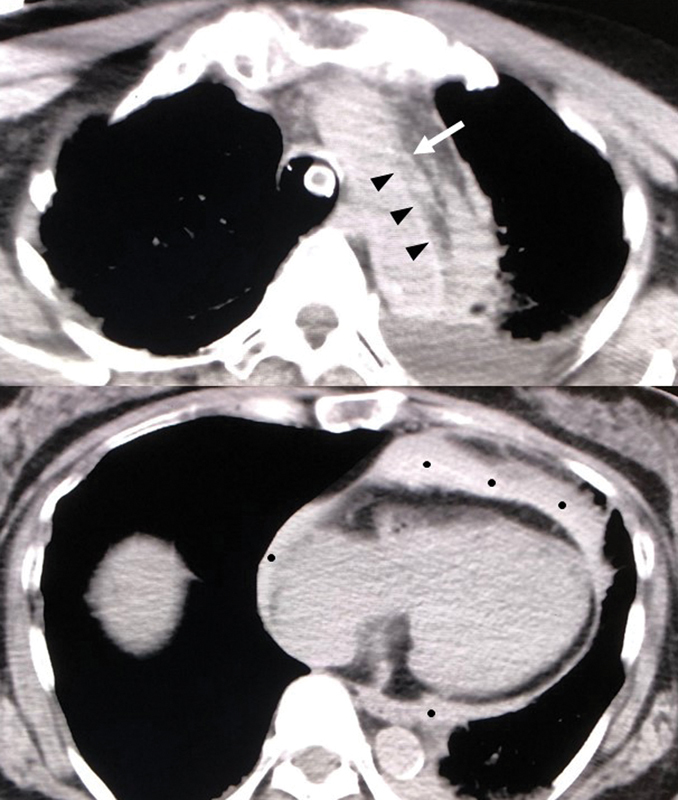
Autopsy imaging. The imaging disclosed Stanford's Type A aortic dissection with cardiac tamponade.

A 74-year-old woman suddenly showed focal convulsion of the right upper extremity and right-side face with right conjugated deviation, following a loss of consciousness in front of her family. Her son called an ambulance. After a few minutes, she regained consciousness and complained of epigastralgia with cold perspiration. Her medical history included hypertension and hyperlipidemia. On arrival, she had clear consciousness, with a systolic blood pressure of 90 mm Hg, a heart rate of 66 beats per minute, and oxygen saturation of 95% under room air. A physical examination that included a neurological examination, chest roentgenography, and electrocardiography (ECG) revealed no specific findings. A blood test revealed the following: white blood cell count, 10,000/μL; hemoglobin, 13.9 g/dL; platelet count, 12.7 × 10 ^4^ /mm ^3^ ; total protein, 6.5 g/dL; glucose, 121 mg/dL; total bilirubin, 0.5 mg/dL; aspartate aminotransferase, 21 IU/L; alanine aminotransferase, 16 IU/L; creatine phosphokinase, 77 IU/L; blood urea nitrogen, 17.2 mg/dL; creatinine, 0.70 mg/dL; sodium, 140 mEq/L; potassium, 4.1 mEq/L; chloride, 104 mEq/L; C-reactive protein, 0.3 mg/dL; activated partial thromboplastin time, 32.5 (26.2) s; international normalized ratio of prothrombin time, 0.98; fibrinogen, 217 mgL/dL; and fibrinogen degradation product (FDP), 377 μg/mL; troponin T was not detected. Head computed tomography (CT) showed normal findings, head magnetic resonance imaging (MRI) showed only old lacunar infarctions, and MR angiography did not show any significant stenosis due to dissection. As her initial complaints subsided spontaneously, the internal physician and neurologist allowed her to return home. However, the next morning, she was found in cardiac arrest by her family. She was transported to our hospital again. Her initial rhythm was asystole and advanced cardiac life support failed to obtain a return of spontaneous circulation. Autopsy imaging revealed Stanford's Type A aortic dissection with cardiac tamponade ( Fig. 1 ). Her family did not permit a classical autopsy.

## Discussion

Focal convulsion as an initial sign of aortic dissection is extremely rare. This was the fourth case reported in the English literature. In the first case, Mo et al 3 reported a 46-year-old man with Type A aortic dissection who experienced a sudden loss of consciousness and right hemiconvulsive movements. 3 On arrival, he had left hemiplegia. As MRI showed an area of abnormal hyperintensity in the right-middle cerebral artery territory on diffusion-weighted imaging, he was initially diagnosed with acute cerebral infarction with convulsion. However, routine chest radiography revealed a widened mediastinum. Reevaluation of the patient revealed significant blood pressure differentials in the patient's arms. Chest CT revealed the Type A aortic dissection. The authors suggested that extension of dissection into the common carotid arteries or through thromboembolism or cerebral hypoperfusion due to Type A dissection might be a possible mechanism underlying seizure as the initial presentation of aortic dissection. In the second case, Oon et al 4 reported a 66-year-old man with two brief periods of jerking movements involving the left upper limb which spontaneously ceased. Head CT showed no fresh lesions; thus, the initial diagnosis was status epilepticus secondary to scar epilepsy from a previous nonhemorrhagic infarct. However, he developed refractory severe hypotension, bradycardia, and hypoxia requiring mechanical ventilation and inotropic support. ECG showed new deep ST-segment depressions in the inferolateral leads and a coronary angiography revealed an aortic root dissection with possible compression of the left main coronary artery. The authors suggested that the sudden onset of symptoms heralded the beginning stages of aortic dissection, with left upper limb seizures resulting from dissection into the right carotid artery and the persistent loss of consciousness from global cerebral hypoperfusion. In the third case, Chen et al 5 reported a 61-year-old male who presented with a transient loss of consciousness accompanied by upward gaze and limb convulsion. Head CT revealed no specific findings. Chest X-ray revealed widening of the mediastinum. Subsequently, chest CT demonstrated Stanford's Type A aortic dissection. The patient experienced a loss of consciousness, accompanied by upward gaze and limb convulsion for approximately 10 seconds in the emergency room, and ECG at that time revealed transient cardiac asystole followed by a spontaneous recovery of the sinus rhythm. Accordingly, they concluded that cardiac asystole had been caused by a painless Type A aortic dissection that led to convulsive syncope because cerebral perfusion had been impeded.

Type A dissection involves the aortic arch, which may result in seizure either through the extension of the dissection into the common carotid arteries or through thromboembolism or cerebral hypoperfusion. 6 Focal seizures may be induced by hypotension based on vulnerability of the previously compromised cerebral tissue or vessels due to changes in blood pressure. 7 In the present case, the patient was in a state of shock on arrival and had old lacunar infarctions in her brain without fresh cerebral ischemia; thus, focal seizure with hypotension (cerebral hypoperfusion) due to Type A dissection was the most likely mechanism in the present case.

The patient showed a high level of FDP on arrival. As she did not have any signs of pulmonary embolism, whole body CT should have been performed to investigate the possibility of deep vein thrombosis, trauma, infection or disseminated intravascular coagulation, aortic dissection, or hematoma. 8 Accordingly, physicians should perform whole body CT to determine whether or not aortic dissection is present when treating patients with convulsion and a high level of FDP.
